# Supplementary material for: Extracellular vesicles in human semen modulate antigen-presenting cell function and decrease downstream antiviral T cell responses
Source: PLoS One. 2019 Oct 17;14(10):e0223901. doi: 10.1371/journal.pone.0223901 (PMC6797208; doi:10.1371/journal.pone.0223901)
Supplement: S1 Fig — Five recipients were tested, error bars indicate standard deviation for technical replicates (A) Monocyte-derived DCs were mock treated or treated with 105 SEV overnight. Cells were stained with live-dead viability stain, fixed, and analyzed by flow cytometry. Cells are gated on high SSC DCs and the percent of live (unstained) cells are plotted on the y-axis. (B) Negatively selected T cells were mock treated or treated with 105 SEV overnight. Cells were stained with live-dead viability stain, fixed, stained with an anti-CD3 antibody and analyzed by flow cytometry. The percent of live CD3+ cells is plotted. (PPTX) [file pone.0223901.s001.pptx]

## Slide 1
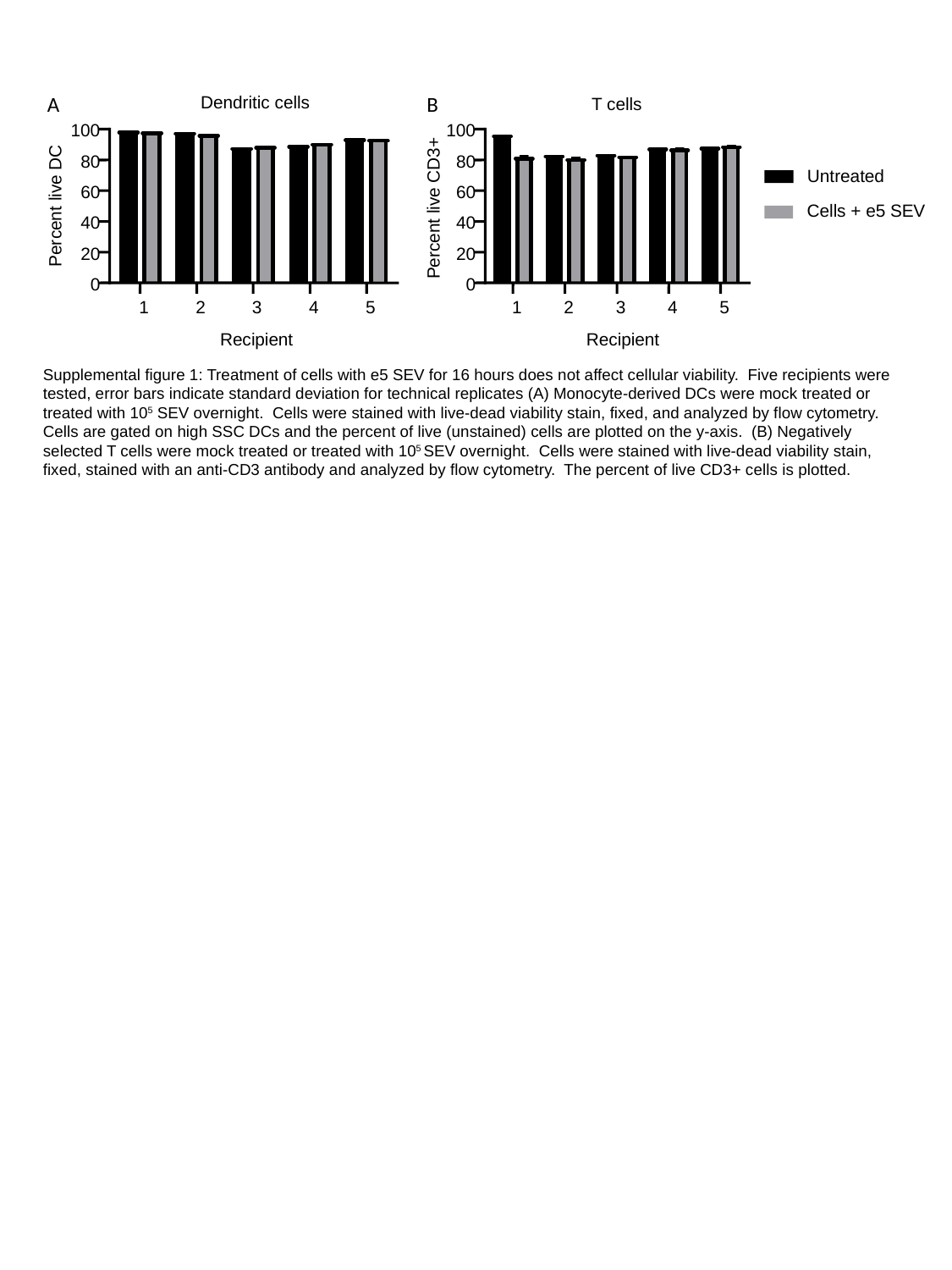

A
B
Dendritic cells
100
80
60
40
20
0
1
2
3
4
5
Recipient
T cells
100
80
60
40
20
0
1
2
3
4
5
Recipient
Percent live CD3+
Untreated
Cells + e5 SEV
Percent live DC
Supplemental figure 1: Treatment of cells with e5 SEV for 16 hours does not affect cellular viability. Five recipients were tested, error bars indicate standard deviation for technical replicates (A) Monocyte-derived DCs were mock treated or treated with 105 SEV overnight. Cells were stained with live-dead viability stain, fixed, and analyzed by flow cytometry. Cells are gated on high SSC DCs and the percent of live (unstained) cells are plotted on the y-axis. (B) Negatively selected T cells were mock treated or treated with 105 SEV overnight. Cells were stained with live-dead viability stain, fixed, stained with an anti-CD3 antibody and analyzed by flow cytometry. The percent of live CD3+ cells is plotted.
